# Supplementary material for: The PI(4)P phosphatase Sac2 controls insulin granule docking and release
Source: J Cell Biol. 2019 Sep 18;218(11):3714–29. doi: 10.1083/jcb.201903121 (PMC6829663; doi:10.1083/jcb.201903121)
Supplement: Supplemental Materials (PDF) [file JCB_201903121_sm.pdf]

Supplemental material

Nguyen et al., https://doi.org/10.1083/jcb.201903121

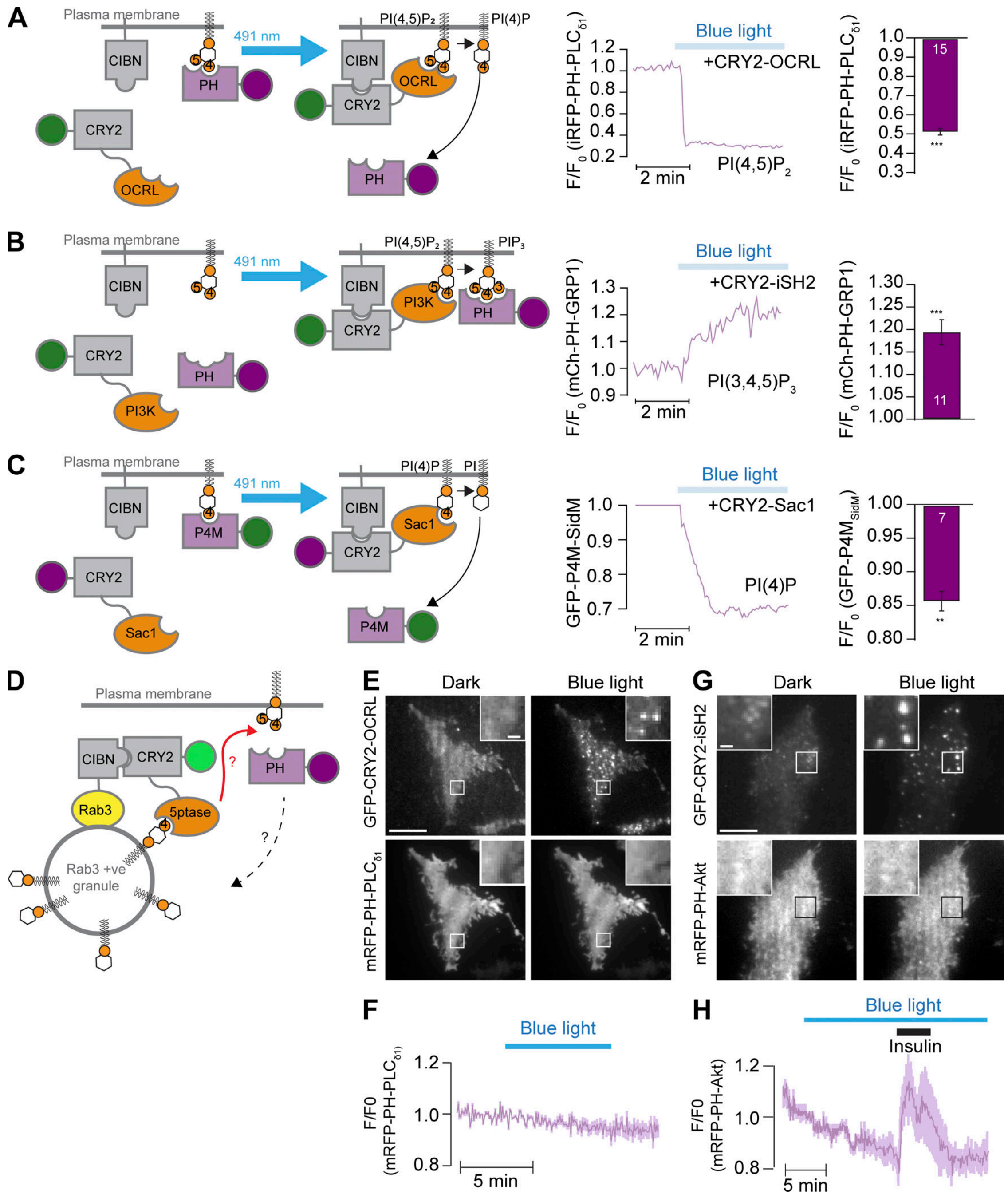

Figure S1. **Optogenetic modulation of phosphoinositides in cellular compartments. (A–C)** Control experiments to assess the substrate specificity of the optogenetic modules used in this study. Illustrations to the left show the principle of light-induced recruitment of phosphoinositide metabolizing enzymes to the plasma membrane. To the right are shown example TIRF microscopy recordings of the effect of recruiting the 5′-phosphatase domain of OCRL on plasma membrane mRFP-$PH_{PLC\delta1}$ fluorescence (PI(4,5)P_2; A), the iSH2 domain from PI3 kinase on plasma membrane mCherry-PH_{GRP1} fluorescence (PI(3,4,5)P_3; B), and the 4′-phosphatase domain of Sac1 on plasma membrane mCherry-$P4M_{SidM}$ fluorescence (PI(4)P; C). Quantifications of the fluorescence change at the plasma membrane (mean ± SEM) following illumination are shown to the left of each trace (number of cells indicated; **, $P < 0.01$; ***, $P < 0.001$, Student's paired *t* test). **(D)** Principle of light-induced recruitment of phosphoinositide-metabolizing enzymes onto Rab3-positive granules. **(E and F)** TIRF micrographs (E) and recording (F) showing the effect of blue-light recruitment of GFP-CRY2-OCRL onto CIBN-Rab3 granules on plasma membrane PI(4,5)P_2 levels (mRFP-$PH_{PLC\delta1}$; $n = 11$ cells). Scale bar is 10 µm (inset, 1 µm). **(G and H)** TIRF micrographs (G) and recording (H) showing the effect of blue-light recruitment of GFP-CRY2-iSH2 onto CIBN-Rab3 granules on plasma membrane PI(3,4,5)P_3 levels (mRFP-PH_{Akt}). The addition of 100 nM insulin results in the formation of PI(3,4,5)P_3 ($n = 12$ cells). Scale bar is 10 µm (inset, 1 µm).

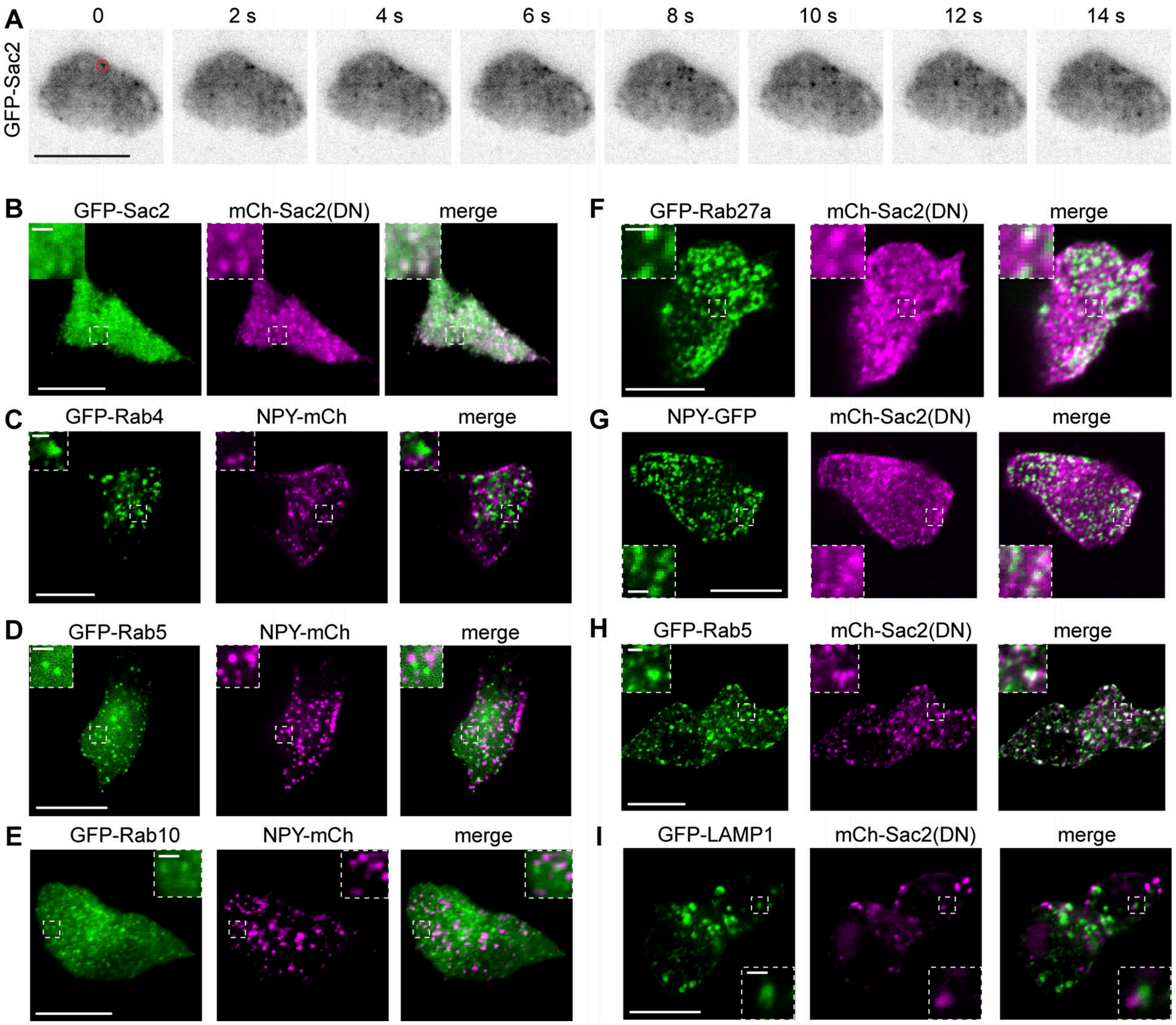

Figure S2. **Colocalization between Sac2 and organelle markers. (A)** TIRF microscopy images of a MIN6 cell expressing GFP-Sac2. Images were taken with 2-s intervals and show punctate structures that are both static (example marked with a circle) and dynamic. Images have been inverted to show fluorescence in black. **(B)** TIRF microcopy images of a MIN6 cell expressing GFP-Sac2 (green) and mCherry-Sac2-DN (magenta). **(C)** Confocal microcopy images of a MIN6 cell expressing GFP-Rab4 (green) and NPY-mCherry (magenta). **(D)** Confocal microcopy images of a MIN6 cell expressing GFP-Rab5 (green) and NPY-mCherry (magenta). **(E)** Confocal microcopy images of a MIN6 cell expressing GFP-Rab10 (green) and NPY-mCherry (magenta). **(F)** Confocal microcopy images of a MIN6 cell expressing GFP-Rab27a (green) and mCherry-Sac2-DN (magenta). **(G)** Confocal microcopy images of a MIN6 cell expressing NPY-GFP (green) and mCherry-Sac2-DN (magenta). **(H)** Confocal microcopy images of a MIN6 cell expressing GFP-Rab5 (green) and mCherry-Sac2-DN (magenta). **(I)** Confocal microcopy images of a MIN6 cell expressing GFP-LAMP1 (green) and mCherry-Sac2-DN (magenta). Scale bars are 10 µm (insets, 1 µm).

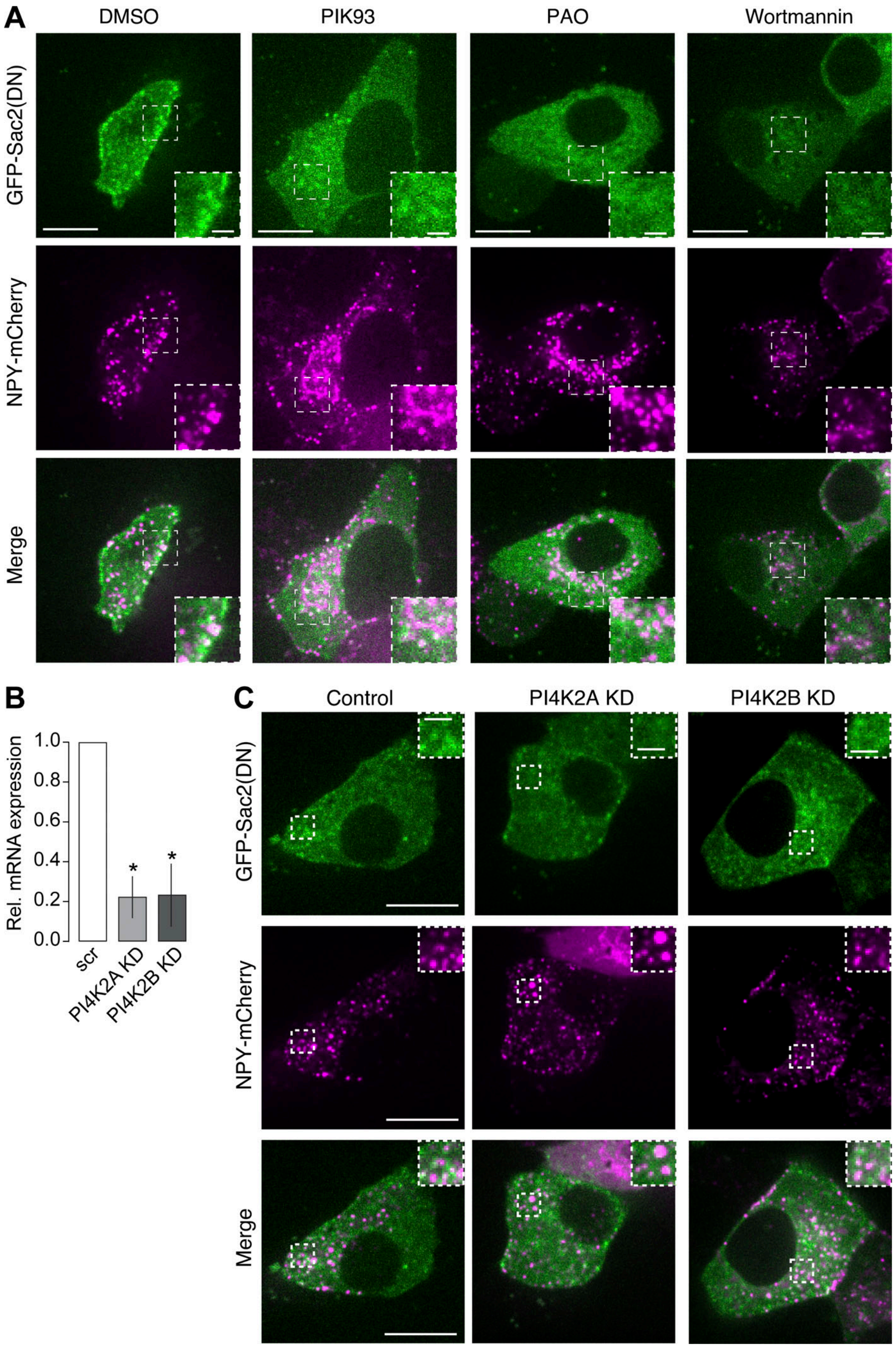

Figure S3. **Involvement of PI4 kinase in the localization of Sac2 on insulin granules. (A)** Confocal microscopy images of MIN6 cells expressing GFP-Sac2-DN (green) and NPY-mCherry (magenta) following treatment with DMSO (1 µl/ml), PIK93 (30 µM), PAO (50 µM), or wortmannin (10 µM). Scale bars are 10 µm (insets, 2 µm). **(B)** RT-PCR measurements of PI4K2A and PI4K2B mRNA levels in control cells and cells treated with the indicated siRNA (mean ± SD for two separate experiments; *, $P < 0.01$ compared with control as assessed by unpaired Student's *t* test). **(C)** Confocal microscopy images of MIN6 cells expressing GFP-Sac2-DN (green) and NPY-mCherry (magenta) following treatment with control siRNA or siRNA targeting PI4K2A or PI4K2B. Scale bars are 10 µm (insets, 2 µm).

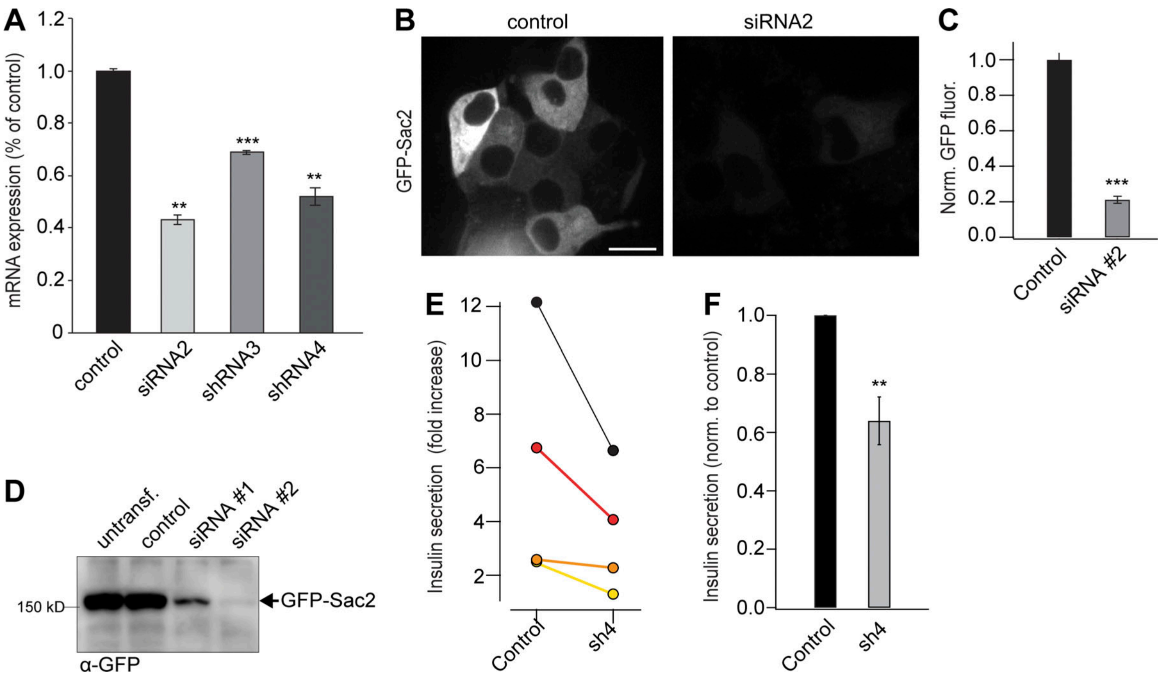

Figure S4. **Sac2 KD suppress glucose-stimulated insulin secretion. (A)** Quantitative RT-PCR analysis of Sac2 mRNA expression in control and Sac2 KD cells (n = 4; **, P < 0.01; ***, P < 0.001, Student's unpaired t test). **(B and C)** Confocal microscopy images of GFP-Sac2 in MIN6 cells treated with control or Sac2 siRNA for 72 h (B). The average fluorescence is quantified in C (mean ± SEM, n = 157 and 158 cells; ***, P < 0.001, Student's unpaired t test). Scale bar is 10 µm. **(D)** GFP immunoblot of lysates from control and Sac2 siRNA–treated MIN6 cells cotransfected with GFP-Sac2. **(E and F)** Insulin secretion (fold change between 20 mM and 3 mM glucose) from control and Sac2 stable KD cells. Data in F are mean ± SEM for four experiments (**, P < 0.01, Student's paired t test).

https://doi.org/10.1083/jcb.201903121

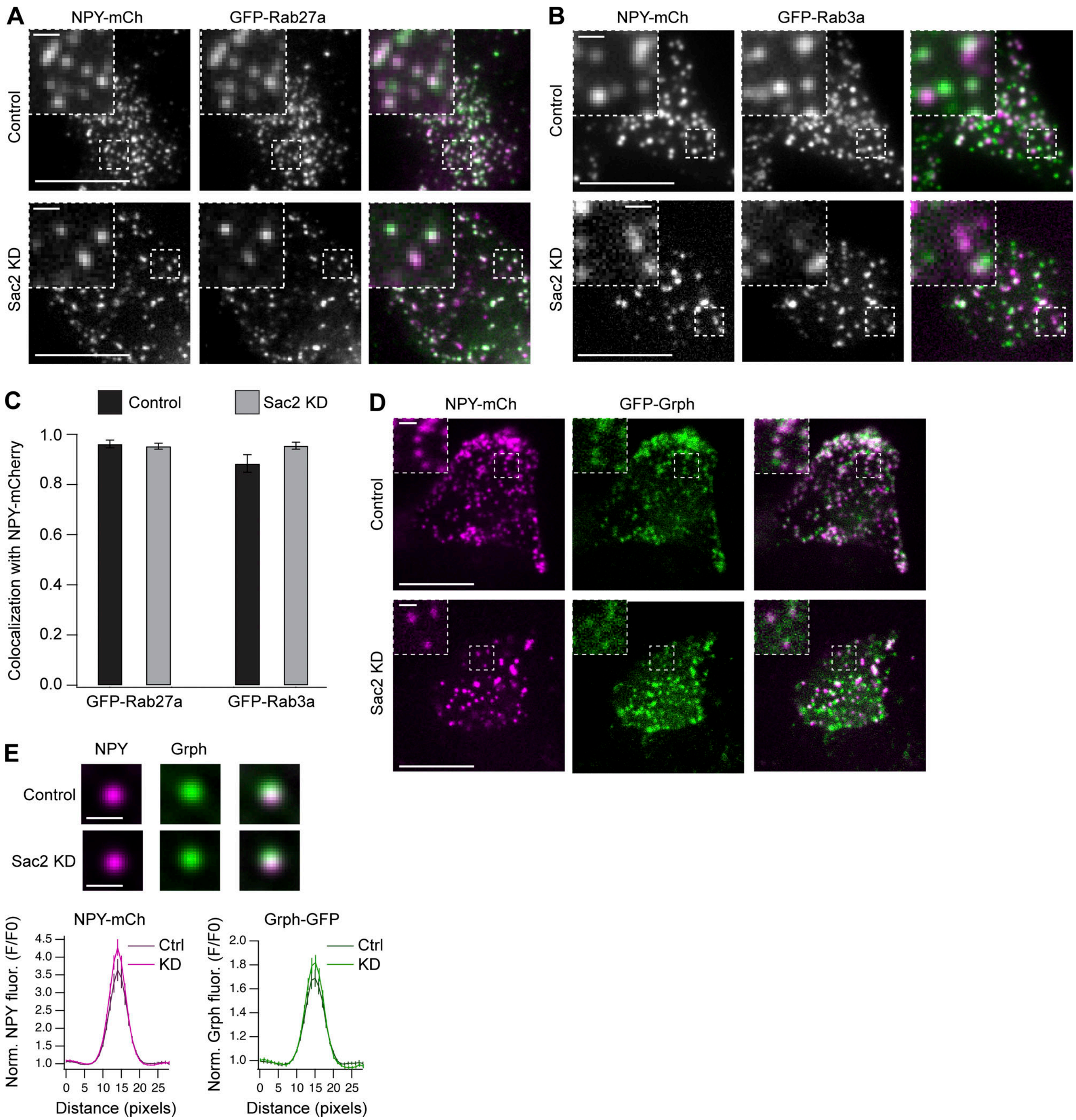

Fig. S5. **Sac2 KD is without effect on Rab3a and Rab27a distribution. (A and B)** Confocal microscopy images of control or Sac2 KD MIN6 cells expressing NPY-mCherry and GFP-Rab27a (A) or GFP-Rab3a (B). Scale bars are 10 µm (insets, 1 µm). **(C)** Quantification of the colocalization of NPY-mCherry with GFP-Rab27a or GFP-Rab3a in control and Sac2 KD cells. Data are presented as mean ± SEM for 10–18 cells. Scale bars are 10 µm (insets, 1 µm). **(D)** Confocal microscopy images of control and Sac2 KD MIN6 cells expressing NPY-mCherry (magenta) and GFP-granuphilin (green). **(E)** Images (top) show the average NPY-mCherry fluorescence (magenta) and the associated GFP-granuphilin fluorescence (green) from control and Sac2 KD cells. Line profiles below are from lines drawn diagonally across the images. Data are presented as mean ± SEM for 23 (control) and 29 (Sac2 KD) cells (15–20 structures per cells). Scale bars are 1 µm.
